# Financial reporting quality of ESG firms listed in China

**DOI:** 10.1371/journal.pone.0284684

**Published:** 2023-06-13

**Authors:** Mengqian Wu, Indra Abeysekera

**Affiliations:** Charles Darwin University, Darwin, Australia; Yunnan Technology and Business University, CHINA

## Abstract

Given the growing importance of environmental protection in China, this study investigated the determinants of the financial reporting quality of environmental, social and governance (ESG) firms listed in China. The quality of financial reporting shows how informative the accounting numbers are for decision-making. Because business outlook can influence financial reporting quality, this study examined predictable, moderately predictable and unpredictable business outlooks. The study randomly selected 100 firms from the 2021 China ESG Top 500 Outstanding Enterprises published by the Sina Finance ESG Rating Centre and then analysed those firms in 2018, 2019 and 2020. It investigated determinants (financial health, governance and earnings management), controlling for the influence of known variables (firm age and firm-specific risk) on financial reporting quality measured as accruals quality and earnings smoothness. Ordinary robust least square regression was conducted. Financial health had a negative influence, but governance variables and earnings management did not affect financial reporting quality. Firm-specific risk had a positive effect, but firm age did not influence financial reporting quality. Changes in business outlook had no impact on the determinants’ effect on financial reporting quality. The study found that ESG firms did not engage in earnings management and aggressively manage earnings, pointing to ethical behaviour. This is the first study to contribute to understanding the financial reporting quality of ESG firms listed in China. It examined different business outlooks to understand ESG firms’ behaviour towards financial reporting quality. The findings invite replicable studies outside China to understand the contextual validity and reliability of the financial reporting quality of ESG firms, and to investigate the effect of determinants not examined in this study.

## 1. Introduction

Environmental, social and governance (ESG)–certified firms follow a set of criteria in project investment that traditional investing excludes from the analysis. The ESG certification criteria apply to three aspects—environmental, social and governance—that show accurate and verifiable results to demonstrate their established journey towards sustainability [1].

Social determinants include working conditions, labour, conflicts, health and safety, employee relations, and diversity. Examples of environmental impacts include climate change, waste and pollution, deforestation, and resource depletion. Factors that determine the quality of governance are executive pay, corruption, board composition, board diversity, board structure, political affiliations and tax strategies [1].

Firms’ qualified investments, considered ethical investing, make them ESG firms. Firms can select their preferred pathways towards ESG certification—for example, the carbon disclosure project, commercial green building and science-based target initiatives. The aspect of ESG that is considered the most important varies between firms and countries [1].

Research has widely studied the financial performance of ESG firms. A review of these studies shows that 90% of ESG firms indicate non-negative financial performance, with many showing a positive association with ESG investments, and are stable over time [2]. Earnings are the most crucial parameter in financial performance and show the result of value-added activities in a reporting period [3].

The ESG scenario in China is unique. The ESG scores assigned to Chinese firms are typically lower than those of their global peers, with many firms ranked below the global median. China ranked 48 out of 50 countries in environmental rankings, with a higher score being worse, mainly because of the low rate of ESG disclosure by Chinese firms [4].

Chinese firms ranked lower on various environmental issues, including carbon emissions, toxic emissions and waste. On the governance side, Chinese firms pay lower remuneration to board members and have a smaller proportion of independent directors. On the social side, Chinese firms face heavier scrutiny because they have lower privacy levels and a higher occurrence of data breaches. These issues make investors sceptical about investing in Chinese ESG firms, which may hinder foreign capital inflows into these firms [4].

There are several perspectives from which to perceive the value creation of ESG firms. ESG firms comprise stakeholders and shareholders to create value. These firms embrace an implicit contract with society to invest in environmentally and socially beneficial projects [5]. However, a much contested issue is that these investing strategies diminish overall shareholder return, making ESG firms subordinate to typical firms [6,7]. An ESG strategy that does not produce favourable financial results can bring financial distress to ESG firms and affect financial reporting quality. It can also encourage ESG firms to engage in earnings management. The systematic risk in the market can change with the business outlook, and the outlook environment can also influence changes in financial reporting quality.

Financial reporting quality, also known as earnings quality, is crucial because it indicates the usefulness of earnings information. Low-quality financial reporting resulting from overstating or understating earnings is undesirable because basing decisions on such data can facilitate the misallocation of resources and result in low financially growth. High-quality earnings, faithfully representing Hicksian income, support decision usefulness. Hicksian income shows the amount consumed in a reporting period and leaves the firm better off than at the beginning of the reporting period, showing the change in the financial worth of the net assets [8,9]. Research has investigated the financial reporting quality of ESG firms influencing various outcomes, but there is a lack of understanding of different determinants influencing financial reporting quality [10].

This study investigates the influence of financial health/distress, earnings management and governance factors on the financial reporting quality of Chinese-listed ESG firms. The study comprises sample firm data from three periods: 2018 (before COVID-19), which represents a predictable business outlook; 2019, which represents a moderately unpredictable business outlook; and 2020, which represents an unpredictable business outlook. Since COVID-19 came to China’s attention in November 2019, businesses have responded cautiously [11]. Hence, this study also investigates whether different business outlooks had a systemic influence on the sample firms.

The next section discusses the relevant literature. Section three outlines the theoretical framework. Section four discusses the methodology. Section five is results. The final section is conclusion.

## 2. Relevant literature

There is a shortage of literature with a focus on the effect of governance, financial health and earnings management on the financial reporting quality of ESG firms, specifically in China.

### 2.1 Financial reporting quality

Early literature examined the measurement of accounting numbers as a guide to financial reporting quality. The focus of these studies was to reach a commonly agreed accounting framework. These studies included measuring earnings by matching costs with revenue [12], replacing historical costs of replacement prices [13], exit costs [14], and discounted cash flows generated by assets and liabilities [15]. Regardless of the measurement differences, the studies assumed that earnings show the underlying firm performance [16,17].

The decision and measurement models influence firms’ reporting about financial performance as earnings. Research has pointed out that earnings results from measuring accounting numbers and to whom firms report them for decision-making. This reveals that financial reporting quality is not a unidimensional construct because it depends on the decision-usefulness model and the accounting measurement model that are used. The quality of earnings showing informativeness is for decision-usefulness [18].

The decision-usefulness model influences the responsiveness of the investor and stakeholder to the reported earnings. The measurement model can accommodate errors and misstatements that decrease earnings accuracy. These models can affect financial reporting quality and therefore the relationship between timely earnings and operating cash flows [18,19].

Financial reporting quality typically comprises four attributes: accrual quality, earnings smoothness, earnings predictability and earnings persistence [20]. The first two attributes relate to the timely relationship between earnings and cash flows. The extent of accruals becoming cash flows (accrual quality) and the extent to which managers use their informed judgement to approximate earnings with operating cash flows (earnings smoothness) are two more attributes. They are crucial for ESG firms, which engage in investments following ESG criteria with a more extended gestation period. ESG firms must convince about cash flow sufficiency using various methods (e.g., dividend payouts strategy) [7,20]. These investments have various unobservable performances relating to the firm’s revenue, expenses, assets and liabilities. Managers must judge investments for reported earnings, considering various stakeholders for longer-term value creation [21]. This study focuses on accrual quality and earnings smoothness in the primary analysis as financial reporting quality attributes.

The two earnings qualities that consider time series are whether they persist from one period to the subsequent periods (earnings persistence) and whether future reported earnings are predictable from current reported earnings (earnings predictability) [22]. Different business outlooks can disrupt the time series earnings and produce unsatisfactory results for earnings predictability. The additional analysis tests for earnings persistence and reports its validity for the ESG firms.

#### 2.1.1 Financial reporting quality attribute—accrual quality

Earnings comprise accruals and cash components. Balance sheets provide current accruals details with inventory changes, accounts receivable, accumulated depreciation, accounts payable, tax payable and other changes in other current assets. The statement of cash flows also provides these items with cash flow impact [23].

Current accruals result from changes in current assets and liabilities; however, there are events related to the firm, but not operating events, that can lead to either overestimating or underestimating accruals, which have a corresponding effect on earnings. For instance, mergers and acquisitions can lead to overestimating accruals, and divestitures can lead to underestimating accruals. Foreign currency translation can depend on the exchange rate applicable at that date and can lead to either over- or underestimating accruals [23].

Studies measure accruals as total accruals by regressing accruals with cash flow from operations in previous, current and future years. However, misjudged accruals do not become cash, and using the balance sheet to find these accruals is likely to produce measurement Type I errors, and studies can then conclude that firms engage in earnings management when they do not [24]. Hence, accruals estimations must aim to decrease these measurement errors to show that the accruals correctly represent future operating cash flows.

Studies can reduce measurement errors by estimating the residual at the firm-level regressions because that represents the unrelated amount, and its standard deviation shows the variation of the unrelated accruals. A higher standard deviation indicates low accrual quality comprising unrelated accruals [25].

#### 2.1.2 Financial reporting quality attribute—earnings smoothness

Managers can use their discretion to use the financial measurement framework to measure earnings to benefit themselves or investors. Studies investigating accounting choices in specific reporting periods define smoothness as the difference between reported earnings and average or typical earnings based on historical estimations [26].

Studies now use a comparative approach between earnings and cash flows to determine earnings smoothness [26,27]. One measure of earnings smoothness is using the standard deviation to show variability, where smoothness is the ratio between the standard deviation of earnings and cash flows from operations. Another measure is the correlation between the change in accruals and the change in cash flows from operations [28].

Firms with more significant cumulative abnormal returns and firms in specific industries smooth earnings more than others, demonstrating that managers can use earning smoothness to increase abnormal returns. Thus, managers show that the firm is more valuable to shareholders, opportunistically influencing the shareholder value [29].

In an alternative approach, firms smooth earnings to make income more meaningful. In this way, managers beneficially influence shareholder value (e.g., operating income after depreciation, pre-tax income, income before extraordinary items, and net income efficient and opportunistic perspectives aim to smooth earnings to report stable earnings across reporting periods [28,29].

#### 2.1.3 Financial reporting quality attribute—earnings persistence

The literature contests that earnings persist across periods as a measure of earnings quality for all firms, stating that it is a firm-specific measure. A strand of research in income smoothing argues that firms that want to report stable income can contribute to persistent earnings [29,30]. Underlying firm performance and accounting measurement rules can also contribute differently to earnings persistence. Research has shown that changes in profit margins (i.e., changes in revenue and expenses) can influence persistent earnings as a result of competition, technology and whether the firm follows a cost leadership or differentiation strategy [27].

#### 2.1.4 Financial reporting quality attribute—earnings predictability

Forecasting earnings based on current earnings benefits internal and external stakeholders. Firms have some flexibility in measuring accounting numbers to report them to stakeholders, which can influence the accuracy of earnings predictability. Research notes that accurate, predictable earnings can assist analysts’ stock forecasts and returns [31]. A study also reports that audited financial statements can produce more accurate forecast earnings [24,32].

### 2.2 Financial health versus financial distress

Financial distress can decrease financial reporting quality as a result of firms manipulating earnings to ease or eliminate the adverse effects resulting from financial distress [15]. Firms undergoing financial distress may want to hide it from investors [26] because it can have negative consequences such as reducing firms’ ability to meet earnings expected by investors, increasing borrowing costs and issuing debts to the market [33,34].

Research has shown that financially distressed Chinese listed firms opportunistically manage earnings, which contributes to decreased financial reporting quality along the four earnings quality attributes: accrual quality, earnings persistence, earnings predictability and earnings smoothness [26,35]. Financially distressed listed firms in China are more likely to engage in accruals-based earnings management [33]. In the Chinese stock market, a firm making losses over three or more successive years leads to suspension or delisting from the stock market. Overregulation can incentivise Chinese firms in financial distress to manipulate earnings [36].

ESG firms can improve their financial health through better financial performance, which can then contribute to improved financial reporting quality [31]. Research indicates that ESG investments and financial performance association occur after several years because ESG investments take time to contribute to financial results [37,38]. However, this conclusion contradicts that of another study, which found that the relationship between ESG investments and financial performance is inconclusive because firms intentionally mislead ESG measurements by using matrices to look good rather than doing good on that front [39]. Firms can exercise discretion in defining and measuring ESG investment–related performance and financial performance. Firms must integrate ESG into their investment strategy rather than screening investments to reject as non-ESG investments. Sustainable firm value creation occurs as a result of improved risk management and by becoming operationally efficient [37,40].

### 2.3 Governance

A corporate governance index that combine all governance variables into a composite index can increase empirical parsimony. However, such indices poorly represent the governance construct. The index can omit important variables and may not include the contextual relevance such as the country’s societal cultural context [41]. More than 80% of Chinese firms have at least one shareholder holding more than 20% of shares. Concentrated ownership structures can decrease the transparent disclosure of corporate governance information to form a publicly available comprehensively represented corporate governance index [42]. Individual governance variables that are selectively chosen and distinctly different from each other can contribute to a more meaningful investigation of the governance construct.

The board of directors is responsible for the firm’s fiduciary functions to ensure that managers work for and safeguard managerial interests [21,42]. As appointees of shareholders, directors reduce agency costs; managers can otherwise take advantage of their position to benefit personally through executive compensations and ownership [43,44].

Directors typically implement an internal control system with policies and procedures that all staff must follow as a monitoring mechanism [7,43]. Internal controls can reduce earnings management using discretionary accruals and making accounting errors, frauds and misstatements ‎[45]. Studies have shown that governance aspects such as board size, independent directors and a diverse board with female directors can enhance internal controls [46,47].

#### 2.3.1 Board size and financial reporting quality

No studies have examined the relationship between large board size and financial reporting quality attributes. However, studies have found that larger boards are associated with earnings management [48–50] Another study has shown that earnings informativeness is negatively related to board size ‎[51], suggesting decreased financial reporting quality.

#### 2.3.2 Independent directors and financial reporting quality

Because executive directors play a significant role in the daily running of the firm, they have a greater understanding of its operations. However, independent directors have little firm-specific knowledge but bring more diversified knowledge gained outside the firm. They are more concerned about their reputation and will likely conduct themselves honestly [52].

#### 2.3.3 Female directors and financial reporting quality

Female directors are more keen to build a trust-based leadership style [39]. They can bring different experiences to the board to enrich board-related discussions, and they have better communication and monitoring skills to improve financial reporting quality [53,54]. Female directors are less supportive of opportunistic behaviours such as (pernicious) earnings management.

### 2.4 Earnings management

There are various definitions of earnings management. It is defined as a purposeful intervention in the external financial reporting process to obtain some personal benefit intentionally [‎45]. Earnings management can occur as a result of the legitimate structuring of transactions, such as the timing of sales and purchase transactions, or illegitimate managerial actions that affect earnings and lead to fraudulent reporting [55–58]. In addition, earnings management can occur when managers use judgement to structure transactions to mislead stakeholders about underlying economic performance or to influence contracts that firms have with accounting numbers [58].

Earnings management is an opportunistic activity; however, there are insufficiently validated arguments for it to be beneficial in providing valuable information [59]. A weak accounting system in China is a contributory factor for earnings management. Accounting standards and their practice development are ad hoc rather than systematically approached. There is evidence that Chinese listed firms commonly practise earnings management, with managers being able to increase their compensation [60].

The literature review indicates extensive literature on financial reporting quality, governance, firm financial health and earnings management. However, there is a lack of research investigating these in relation to ESG firms, and even less about ESG firms in China, with little understanding of the influence of governance, financial health and earnings management on financial reporting quality.

## 3. Theoretical framework

**Fig 1:**
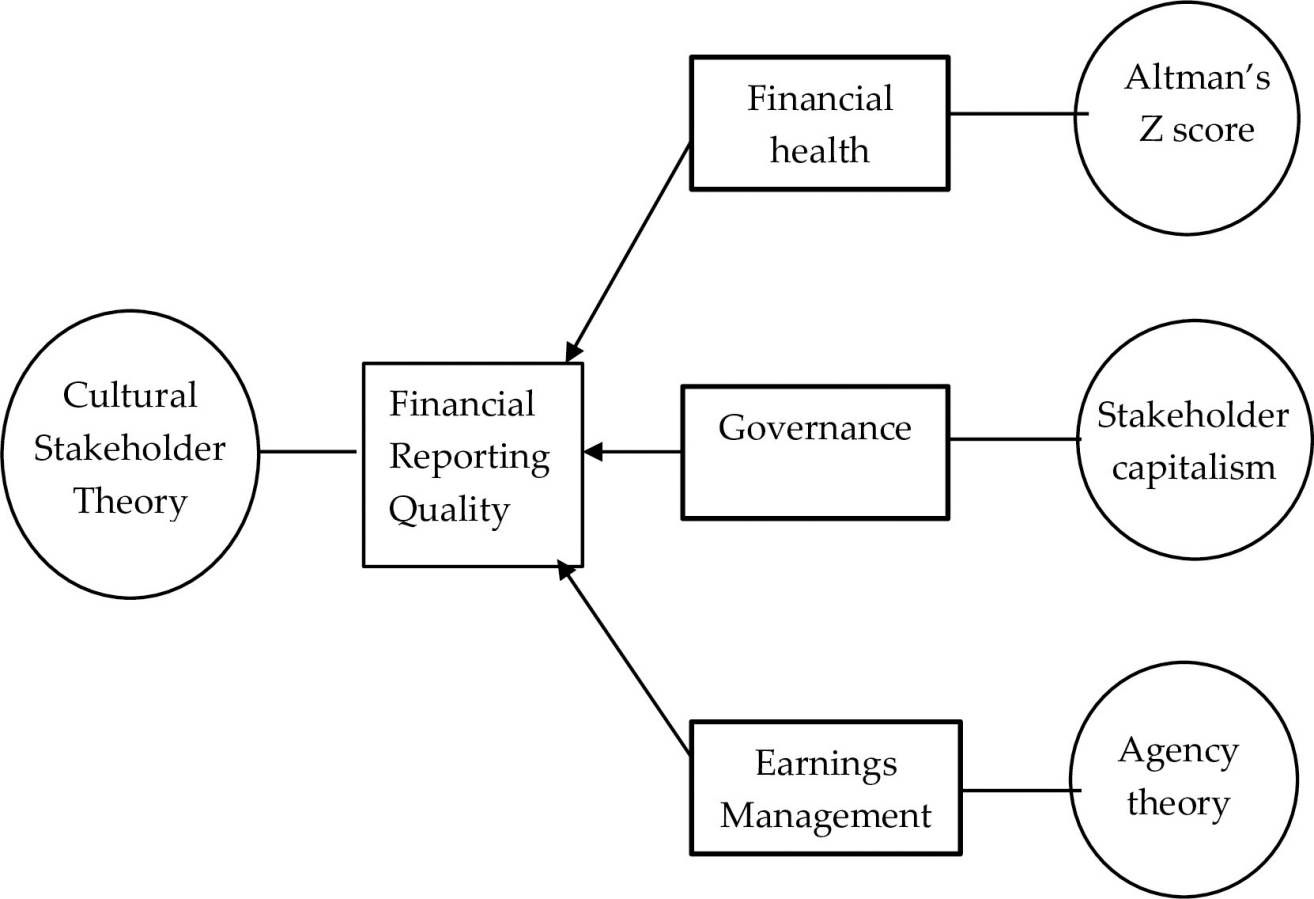
Theoretical framework.

Fig 1 shows the theoretical frameworks related to the study. Four theoretical perspectives are used to explain the financial reporting quality of ESG firms, which have a social reputation. Research has reported that firms with a social reputation are perceived as trustworthy by shareholders and stakeholders but must be evaluated in the context of the societal culture. The cultural stakeholder theory states that the underlying societal cultural conditions can create a mutual relationship between firms and shareholders and other stakeholders [19]. The theory provides a platform to appreciate ESG firms’ financial reporting quality in the context of societal culture in China. Altman’s Z model explains financial health versus distress, stakeholder focus capitalism explains governance, and agency theory explains earnings management.

### 3.1 Financial health versus distress

Altman’s Z-score is a well-established empirical model that examines a firm’s financial health or distress. It was initially developed to investigate manufacturing firms in the United States. The emerging markets score is the revised version with broad applicability, and it has been tested and validated for China [61]. Financial distress occurs when firms experience a shortfall in cash flows, which increases the liquidity risk. This can occur as a result of poor operating performance, high financial leverage and a lack of technological innovation. Asset or financing restructuring is often required to develop viable business models to continue the business [61].

Research has expressed mixed conclusions about ESG firms’ performance. One study showed that ESG funds are not making sufficient earnings because those ESG firms try to maximise stakeholder value through investments in social and environmental projects that are underperforming financially [62]. Yet a literature review reported that ESG investments in firms had a positive association with financial performance beyond the current financial year [3,63,64]. This may be because ESG investments do not decrease financial performance; however, there is a delay in obtaining financial gains from environmental and social investments [64]. ESG firms attempt to maximise the immediate shareholder value, a practice which is prevalent with financially healthy firms [65]. Based on the above discussion, the study states the following hypotheses:

Hypothesis 1: Firm financial health is associated with accrual quality.
Hypothesis 2: Firm financial health is associated with earnings smoothness.

### 3.2 Governance

Corporate governance is a system for directing and controlling firms [42]. In 2019, the Business Roundtable, which is an association of influential chief executive officers, announced a revised corporate commitment to deliver firm value beyond shareholders, identifying other stakeholders as employees, customers, suppliers and communities. This was heralded as a significant milestone of revised purposeful orientation [66].

Following that announcement, the World Economic Forum issued a manifesto asking firms to replace shareholder-focused capitalism with stakeholder-focused capitalism, but it did not specify the stakeholder groups [67]. It has allowed firms to decide who stakeholders are—for example, employees, customers, suppliers, creditors, local communities, society, the environment, the economy of the state or nation, and catch-all categories [67]. Further, capitalism comprising economically and politically designed and propelled private ownership can take different forms, thereby adding to various stakeholder categorisations.

Corporate governance in firms can include stakeholder concerns in making strategic decisions, addressing their concerns and contributing to stakeholders’ capital [68]. One perspective is that the board of directors can decrease its accountability towards shareholder protection, thereby allowing senior executives to maximise their gain and making stakeholder governance a façade for senior executives. An alternative perspective is that senior executives consciously use some of the shareholder value to create value for chosen stakeholders at their discretion [69,70].

Both perspectives show diminishing shareholder value. ESG firms have more investments in social and environmental aspects that can decrease shareholder value to increase stakeholder value. Functioning boards will help improve the quality of financial reporting using this structural approach to augment stakeholder value.

Therefore, this study argues that the governance variables—board size, proportion of independent directors and percentage of female directors—do not influence the financial reporting quality attributes of accrual quality and earnings smoothness. The study states the following hypotheses for the association between governance variables and accrual quality, and between governance variables and earnings smoothness:

Hypothesis 3a: The board size is not associated with accrual quality.
Hypothesis 3b: The proportion of independent directors is not associated with accrual quality.
Hypothesis 3c: The proportion of female directors is not associated with accrual quality.
Hypothesis 4a: The board size is not associated with earnings smoothness.
Hypothesis 4b: The proportion of independent directors is not associated with earnings smoothness.
Hypothesis 4c: The proportion of female directors is not associated with the accrual quality.

### 3.3 Discretionary and nondiscretionary accruals

Discretionary and nondiscretionary accruals are the total accruals that are decomposable. Business activities provide discretion for accruals resulting from investment (e.g., plant, property and equipment depreciation). Business activities require nondiscretionary accruals resulting from revenue, where increased revenue needs increased accounts receivable or inventory.

A firm can aggressively interpret principle-based accounting standards and the generally accepted accounting principles to decide upon accruals. Interpretations can give rise to costly contracts or using accounting information to mislead stakeholders. Discretionary accruals are not easily noticeable from financial statements; models must calculate them [2].

Research has shown that management can easily manipulate current discretionary accruals [71]. They are less likely to engage in earnings management using discretionary or nondiscretionary earnings management. However, there is a higher level of business ethics with operational and strategic ESG objectives. Hence, this study proposes the following hypotheses for the association between earnings management and accrual quality, and between earnings management and earnings smoothness:

Hypothesis 5a: Discretionary accruals are not associated with accrual quality.
Hypothesis 5b: Nondiscretionary accruals are not associated with accrual quality.
Hypothesis 6a: Discretionary accruals are not associated with earnings smoothness.
Hypothesis 6b: Nondiscretionary accruals are not associated with earnings smoothness.

## 4. Methodology

### 4.1 Sample selection

This study randomly selected 100 ESG firms from the 2021 China ESG outstanding enterprises compiled and jointly released by the Sina Finance ESG Rating Centre and CCTV-1 Brands of Great Power program to represent that sample randomly [6,72].

One hundred randomly selected firms sufficiently represent the sample size (greater than 84 firms) for the 500 ESG listed firm population size. The sample was determined by estimating a 0.5 proportion of the population expected to represent the attributes, with a standard error of 0.05 can vary from the true value [73].

The non-randomised error in regression models can trigger endogeneity in field research because such an error term can contain unobserved variables that can affect the sample observations. However, randomly selected firms can randomise the sample’s unobserved variable effect, thus requiring no endogeneity tests [72,74,75]. A random firm selection in a field study is correct modelling that assures causal inference because it meets the orthogonality assumption made by ordinary least squares regression to the treatment condition [28]. Additionally, the study uses the model as a predictive model; which is another reason is that endogeneity testing is not necessary [76].

Table 1 shows that manufacturing firms, followed by construction firms, dominate the industry composition. The sample firms were investigated for 2018, 2019 and 2020. The study excluded nine firms in 2018, three in 2019 and two in 2020. Two firms in the financial industry with ESG scores of zero could not provide data applicable to 2018, 2019 and 2020. Seven firms were listed later than 2018, excluding nine in 2018; thus, there were 91 firms for 2018. The sample listed one firm later than 2019, excluding three firms; thus, there were 97 firms for 2019. Two firms in 2020 had insufficient data; thus, there were 98 firms for 2020. The excluded firms had inadequate data to conduct firm-specific regressions because they required five years of past, current and future year data.

**Table 1:** Distribution of sample firms.

Industry sector	2018	2019	2020
Agriculture, forestry, animal husbandry and fishery	1	1	1
Construction	12	12	12
Culture, sports and entertainment	2	2	2
Information transmission, software and information technology services	3	5	5
Leasing and business services	1	1	1
Manufacturing	63	67	68
Mining	1	1	1
Real estate	1	1	1
Scientific research and technical service	1	1	1
Transportation, warehousing and postal	2	2	2
Water conservancy, environment and public facilities management	1	1	1
Wholesale and retail trade	3	3	3
Total	91	97	98

### 4.2 Data collection

The study obtained data from the China Stock Market & Accounting Research (CSMAR) database and firms’ annual reports. No human participants, human specimens or tissue, vertebrate animals or cephalopods, vertebrate embryos or tissues were involved, nor field research requiring ethics approval.

#### 4.2.1 Dependant variable

Two dependent variables represented financial reporting quality in this study: accrual quality and earnings smoothness.

*Accrual quality*. The dependent variable is total current accruals. This variable measures working capital–based accruals relating to earnings and is calculated by the accruals estimated with firm-specific past five-year, current year and future year rollover regressions.

The total current accrual represents the difference between earnings-based working capital and the change in cash. The previous end-of-the-year total assets standardises the independent variables in the regression equation. The residual in the regression model identifies the unexplained difference between the dependent and independent variables [17].

A higher residual value means lower accrual quality, as shown by the standard deviation of the residual. After ascertaining the accrual quality, the study inverted the resididual value by turning it into a percentile using the equation [(1/x)*100], where x is the firm-specific standard deviation of the residual, to interpret the accrual number conversely. A higher number means higher accrual quality. The CSMAR database provided the financial numbers for the accrual quality regression [17]. The regression equation calculates accrual quality with a five-year r of observations to the current year, as shown in Eq (1):

$$TCA_{t}=[\left(\Delta CA_{t}-\Delta CL_{t}-\Delta Cash_{t}+\Delta STDEBT_{t}+\Delta \;TP_{t}\right)]/TA_{t-1}$$ (1)

Where:

TCA is total current accrual;

ΔCA is the change in current assets from the past year to the current year;

ΔCL is the change in current liabilities from the previous year to the current year;

ΔCash is the change in cash from the prior year to the current year;

ΔSTDEBT is the change in short-term debt from the last year to the current year;

ΔTP is the change in taxes payable from the previous year to the current year;

TA_t−1_ is the total assets at the end of the last year;

t is 2018, 2019 or 2020.

*Earnings smoothness*. This study calculates the second dependent variable—earnings smoothness—by cash flows from operations to earnings both in the current period and standardised by total assets at the end of the previous period. The regression equation calculates earnings smoothness with a five-year r of observations to the current year, as shown in Eq (2). The standard deviation of the calculation shows the variations. A higher number indicates more earnings smoothness, whereby managers aim to inform about cash flows from operations using the earnings information calculated using the following equation [23]:

$$ESt=(\sigma (CFO_{t}/TA_{t-1}))/(\sigma (Earn_{t}/TA_{t-1}))$$ (2)

Where:

ES is the earnings smoothness;

CFO is the cash flows from operations;

Earn is the earnings before taxes;

TA_t−1_ is the total assets at the end of the previous year;

σ is the standard deviation;

t is 2018, 2019 or 2020.

#### 4.2.2 Independent variables

This study has three independent variable strands: financial distress, governance and earnings management. Firms’ annual reports and the CSMAR database provided the data for these variables.

*Financial distress*. This study uses the emerging market score (EMS) predictive model to measure financial distress by combining four different financial ratios. The study calculates the financial ratios using data provided by the CSMAR database. The EMS score calculates the financial distress. A score of less than 1.23 indicates that a firm is heading towards bankruptcy, and a financially healthy firm has a score above 2.9 [77]. The EMS score has been tested across industries and countries, including China, and found to be a reliable index. It is calculated using the EMS regression with the past four years and the current year, as shown in Eq (3) [78,79]:

$$EMS_{t}=6.56*X1_{t}+3.26*X2_{t}+6.72*X3_{t}+1.05*X4_{t}+3.25$$ (3)

Where:

EMS is the emerging markets score;

X1 is the working capital/total assets;

X2 is the retained earnings/total assets;

X3 is the earnings before interest and tax (EBIT)/total assets;

X4 is the book value of equity/total liabilities;

t is 2018, 2019 or 2020.

*Governance variables*. Board size is the total number of directors on the board in each sample year obtained from annual reports. The study calculates the percentages of independent directors as the percentage of number of independent directors divided by the total number of directors using data obtained from annual reports. Annual reports also provided data about independent and female directors.

*Earnings management*. Jones’s earnings management calculation model assumes that variations in revenue can cause nondiscretionary accruals. However, credit sales can add to discretionary accruals through accounts receivable [42]. The modified Jones model used here recognises this in determining the nondiscretionary accruals [26]. The study calculates nondiscretionary accruals using the regression equation on firm-specific past four-year and current-year observations. The predicted value of the regression equation is nondiscretionary accruals, and the standard residual from the residual output of the regression is discretionary accruals [27], as shown in Eq (4):

$$NDA_{t}=\alpha_{1}(1/TA_{t-1})+\alpha_{2}(\Delta REV_{t}‐\Delta REC_{t})+\alpha_{3}PPE_{t}$$ (4)

Where:

NDA_t_ is nondiscretionary accruals;

PPE_t_ is property plant and equipment;

t is 2018, 2019 or 2020.

#### 4.2.3 Control variables

Two control variables are used in this study: firm-specific risk and firm age. Firm-specific risk is the variation in the return on assets in the past four years and current year observations, as shown in Eq (5) [79]. Research has shown that firm-specific risk is associated with earnings management and can influence financial reporting quality [71,79,80]. It is vital to control for unsystematic risk of ESG firms, because this study investigates the influence of systematic risk arising from business outlooks. Company age is the difference between the sample year and its incorporation year [71]. Company age has provided mixed evidence regarding its influence on earnings quality [61,62], and including it can provide further clarity in the context of ESG firms in China. These data were obtained from the CSMAR database.


$${\mathrm{ROA}}_{t}/\mathrm{standard}\;\mathrm{deviation}\;\mathrm{of}\;{\mathrm{ROA}}_{t}$$ (5)

Where:

ROA_t_ is the return on assets;

t is 2018, 2019 or 2020.

### 4.3 Regression model equations

Three separate regressions are conducted for 2018, 2019 and 2020. The study uses two ordinary least square regressions with the robust function: one for the accrual quality, as shown in Eq (6), and the other for earnings smoothness, as shown in Eq (7). The ordinary least squares linear regression is appropriate for this study because the sample obtained is random, and sufficient observations support the number of parameters estimated to make the model parsimonious. There is no multicollinearity and the robust function is used to correct for heteroscedasticity. Satisfying these noted assumptions ensures that the model relationship between the dependent variable and independent variables minimises the sum of squares errors between the actual values and the predicted values of the dependent variable included in the regression model [81].


$$AQ_{t}=a+b_{1}\;EMS_{t}+b_{2}\;BS_{t}+b_{3}\;\%ID_{t}+b_{4}\;\%FD_{t}+b_{5}\;\%DAC_{t}+b_{6}\;\%NDAC_{t}+c_{1}\;\%AGE_{t}+c_{2}\;\%RISK_{t}$$ (6)

$$ES_{t}=a+b_{1}\;EMS_{t}+b_{2}\;BS_{t}+b_{3}\;\%ID_{t}+b_{4}\;\%FD_{t}+b_{5}\;\%DAC_{t}+b_{6}\;\%NDAC_{t}+c_{1}\;\%AGE_{t}+c_{2}\;\%RISK_{t}$$ (7)

Where:

AQ is the accrual quality;

ES is the earnings smoothness;

EMS is the firm-specific emerging markets score;

BS is the board size;

ID is the independent directors in a firm;

FD is the female directors in a firm;

DAC is the firm-specific discretionary accruals;

NDAC is the firm-specific nondiscretionary accruals;

AGE is the age from incorporation year to sample year;

RISK is the firm-specific risk;

t 2018, 2019 or 2020.

## 5. Results

### 5.1 Descriptive statistics

Tables 2–4 summarise the descriptive statistics. The values were similar for the given variables across the three years. Across the sample firms, there was a wide range of minimum and maximum values for accrual quality (AQ). The mean value was greater than the median value, showing that a typical firm had an accrual quality lower than the average firm in the sample. Earnings smoothness (ES) had low mean and median values, showing that managers used discretion. The study used regressions with robust functionality to correct any suspected heteroscedasticity.

**Table 2:** Descriptive statistics of 2018.

Variables	Obs	Max	Min	Median	Mean	SD
***Dependent variables***						
AQ	91	7.951	0.001	0.439	1.015	1.635
ES	91	56.045	0.275	1.633	4.208	7.679
***Control variable***						
AGE	91	63	6	20	20.220	8.312
FSR	91	28.930	−1.930	3.16	4.938	5.352
***Independent variables***						
EMS	91	15.140	2.520	6.83	7.277	2.486
BS	91	15	5	9	8.714	1.990
%FD	91	0.600	0.000	0.13	0.146	0.133
%ID	91	0.800	0.300	0.375	0.394	0.079
DAC	91	1.506	−1.484	0.062	0.056	0.769
NDAC	91	0.160	−0.368	−0.007	−0.009	0.064

**Table 3:** Descriptive statistics of 2019.

Variables	Obs	Max	Min	Median	Mean	SD
***Dependent variables***						
AQ	97	6.967	0.042	0.411	0.938	1.432
ES	97	33.072	0.257	1.588	3.638	5.421
***Control variables***						
AGE	97	64	7	21	21.227	9.233
FSR	97	29.160	−1.730	3.18	5.261	5.460
***Independent variables***						
EMS	97	15.660	3.500	6.87	7.476	2.492
BS	97	15	5	9	8.649	2.082
%FD	97	0.600	0.000	0.13	0.148	0.134
%ID	97	0.800	0.300	0.375	0.397	0.082
DAC	97	1.671	−1.558	−0.262	−0.197	0.833
NDAC	97	0.425	−1.043	−0.017	−0.024	0.129

**Table 4:** Descriptive statistics of 2020.

	Obs	Max	Min	Median	Mean	SD
***Dependent variable***						
AQ	98	7.235	0.039	0.416	0.888	1.371
ES	98	27.019	0.272	1.637	3.160	4.412
***Control variables***						
AGE	98	65	8	22	22.276	9.197
FSR	98	43.750	−1.380	3.71	5.327	6.404
***Independent variables***						
EMS	98	16.050	3.240	7.015	7.502	2.539
BS	98	15	5	9	8.633	2.012
%FD	98	0.600	0.000	0.11	0.143	0.134
%ID	98	0.800	0.286	0.375	0.398	0.087
DAC	98	1.638	−1.558	−0.086	−0.102	0.869
NDAC	98	0.174	−0.240	−0.018	−0.021	0.064

Abbreviations for Tables 2–4, where

AQ is the accrual quality

ES is the earnings smoothness

AGE is the firm age

FSR is the firm-specific risk

EMS is the emerging market score

BS is the board size

%FD is the percentage of female directors on the board

%ID is the percentage of independent directors on the board

DAC is the discretionary accruals

NDAC is the nondiscretionary accruals.

The smoothed earnings had less noise and were more informative [82–85]. The average firm age was around 12–13 years, and the age range in the 2018 sample was 6–63 years. The typical age of a firm was 20 years. The median firm-specific risk in 2018 was 3.16, and the average was 4.9, showing that the sample comprised firms with low firm-specific risks. Some firms made losses, showing negative numbers.

The EMS minimum was over 2, the median was 6.83 and the average was 7.3, showing that the firms were financially healthy. The typical and average board size was seven board members. The median value was, 37% of the total directors were independent directors; the mean value was 40%. The median and means of discretionary accruals were positive, and nondiscretionary accruals were negative.

### 5.2 Correlation with accrual quality

#### 5.2.1 2018 predictable business outlook

Table 5 presents the correlation table for accrual quality. Financially healthy firms indicated by EMS were significantly and negatively associated with AQ. Firm-specific risk (FSR) was significantly and positively associated with AQ, showing firms with more volatile accounting returns contributed to AQ. Larger boards (BS) were significantly and negatively associated with the %ID in all three years. The %FD had a positive association with healthy firms (EMS) in 2018 only.

**Table 5:** Correlation table for accrual quality in 2018.

	AQ	Age	EMS	BS	%FD	%ID	FSR
AQ	1						
AGE	−0.1407	1					
EMS	−0.3800***	0.0141	1				
BS	−0.0346	0.165	0.0385	1			
%FD	−0.1263	0.0804	0.2095**	−0.1628	1		
%ID	0.1081	−0.1355	−0.147	−0.4347***	−0.0973	1	
FSR	0.6762***	−0.0302	0.0018	0.118	−0.0632	−0.0032	1

#### 5.2.2 2019 moderately unpredictable business outlook

Table 6 presents the correlation table for the 2019 output. As reported in 2018, financially healthy firms (EMS) were significantly and negatively associated with AQ, larger boards (BS) had less %ID, and older firms (AGE) had larger boards (BS) and less %ID.

**Table 6:** Correlation table for accrual quality in 2019.

	AQ	Age	EMS	BS	%FD	%ID	FSR
AQ	1						
AGE	−0.1391	1					
EMS	−0.3976***	0.046	1				
BS	−0.0776	0.2096**	0.0042	1			
%FD	−0.1262	0.0413	0.1654	−0.1174	1		
%ID	0.1604	−0.2160**	−0.1814*	−0.4538***	−0.1255	1	
FSR	0.6036***	−0.017	0.0546	0.0683	−0.0139	0.0669	1

**5.2.3 2020 highly unpredictable business outlook.** Table 7 presents the correlation table. As reported in 2018 and 2019, EMS showed a positive association with AQ, older firms (AGE) had a positive association with larger boards (BS), %ID was negatively related to firm age (AGE) and %ID had a negative correlation with %FD.

**Table 7:** Correlation table for accrual quality in 2020.

	AQ	Age	EMS	BS	%FD	%ID	FSR
AQ	1						
AGE	−0.1135	1					
EMS	−0.3666***	0.0285	1				
BS	−0.0203	0.2105**	−0.0539	1			
%FD	−0.0885	0.0513	0.0851	0.0001	1		
%ID	0.1514	−0.2112**	−0.175*	−0.4217***	−0.2590**	1	
FSR	0.6411***	−0.012	0.0194	0.1279	−0.0274	0.054	1

### 5.3 Regression results relating to accrual quality

The study conducted stepwise regression in 2018 (a year with a predictable business outlook), 2019 (a year with a moderately unpredictable business outlook) and 2020 (a year with an unpredictable business outlook).

The first stepwise regression included control variables only, the subsequent stepwise regression added a financial stress variable, the third included governance variables and the final regression model included discretionary and nondiscretionary accrual variables. The variance inflation factor (VIF) was less than 5 in all empirical models, suggesting no correlation between the independent variables and no inflated regression coefficients because of multicollinearity [60].

Tables 8–10 show the AQ stepwise regression results. The stepwise regression conducted in 2018, with a predictable business outlook, showed that FSR significantly and positively influenced AQ. EMS was significantly and negatively associated with AQ. The governance variables had no significant influence on AQ. Firms had not engaged in earnings management, with NDA and DA showing no significant association.

**Table 8:** Stepwise regression for accrual quality in 2018.

	Control variables	Plus, financial health	Plus, governance	Plus, earnings management
***Control variables***				
AGE	−0.024*	−0.023*	−0.020	−0.019
FSR	0.205***	0.206***	0.209***	0.203***
***Independent variables***				
EMS		−0.250***	−0.246***	−0.258***
BS			−0.069	−0.065
% ID			0.089	0.270
%FD			−0.124	−0.138
NDA				2.068
DA				0.095
***Statistic***				
VIF	N/A	1	1.15	1.17
Adjusted R^2^	0.472***	0.616***	0.623***	0.629***
# Observations	91	91	91	91

Notes: ***, ** and * indicate significance at 0.01, 0.05 and 0.1.

**Table 9:** Stepwise regression for accrual quality in 2019.

	Control variables	Plus, financial stress	Plus, governance	Plus, earnings management
***Control variables***				
AGE	−0.020**	−0.017*	−0.014	−0.014*
FSR	0.158***	0.164***	0.167***	0.167***
***Independent variables***				
EMS		−0.245***	−0.244***	−0.247***
BS			−0.088*	−0.0816
% ID			−0.784	−0.560
% FD			−0.685	−0.523
NDA				0.475
DA				−0.102
***Statistic***				
VIF	N/A	1	1.17	1.18
Adjusted R^2^	0.381***	0.562***	0.576***	0.581***
# Observations	97	97	97	97

Notes: ***, ** and * indicate significance at 0.01, 0.05 and 0.1.

**Table 10:** Stepwise regression for accrual quality in 2020.

	Control variables	Plus, financial health	Plus, governance	Plus, earnings management
***Control variables***				
AGE	−0.016*	−0.014	−0.011	−0.012
FSR	0.137***	0.139***	0.142***	0.137***
***Independent variables***				
EMS		−0.203***	−0.208***	−0.233***
BS			−0.084	−0.084
% ID			−0.477	−0.852
%FD			−0.426	−0.335
NDA				2.703
DA				−0.052
***Statistic***				
VIF	N/A	1	1.17	1.21
Adjusted R^2^	0.42***	0.564***	0.577***	0.592***
# Observations	98	98	98	98

Notes: ***, ** and * indicate significance at 0.01, 0.05 and 0.1.

In the four stepwise regressions, the model explanation (adjusted R^2^) increased from the first to the second model after including the financial stress variable showing more informativeness. After that, in model three (with governance variables) and model four (with additional discretionary and nondiscretionary variables), although found to have no significant association with governance variables, and earnings management variables, the model explanation increased. Thus, these variables added to the informativeness of ESG financial reporting quality.

### 5.4 Correlation with earnings smoothness

Tables 11–13 show the pairwise correlation results. EMS showed a negative association with ES. Thus, as firms become more financially healthy, they are less likely to engage in ES. FSR showed a positive association with ES. Thus, firms with more accounting earnings volatility engage in ES. The %FD showed a negative association with EMS, and larger boards (BS) showed a negative association with %ID. The variables showed similar associations with ES and AQ.

**Table 11:** Correlation table for earnings smoothness in 2018.

	ES	AGE	EMS	BS	%FD	%ID	FSR
ES	1						
AGE	−0.1024	1					
EMS	−0.2492**	0.0141	1				
BS	−0.0389	0.165	0.0385	1			
%FD	−0.1016	0.0804	0.2095**	−0.1628	1		
%ID	0.0456	−0.1355	−0.147	−0.4347***	−0.0973	1	
FSR	0.4499***	−0.0302	0.0018	0.118	−0.0632	−0.0032	1

Notes: ***, ** and * indicate significance at 0.01, 0.05 and 0.1.

**Table 12:** Correlation table for earnings smoothness in 2019.

	ES	AGE	EMS	BS	%FD	%ID	FSR
ES	1						
AGE	−0.133	1					
EMS	−0.2732***	0.046	1				
BS	−0.1041	0.2096**	0.0042	1			
%FD	−0.1245	0.0413	0.1654	−0.1174	1		
%ID	0.1578	−0.2160**	−0.1814*	−0.4538***	−0.1255	1	
FSR	0.4761***	−0.017	0.0546	0.0683	−0.0139	0.0669	1

Notes: ***, ** and * indicate significance at 0.01, 0.05 and 0.1.

**Table 13:** Correlation table for earnings smoothness in 2020.

	ES	AGE	EMS	BS	%FD	%ID	FSR
ES	1						
AGE	−0.1073	1					
EMS	−0.2833***	0.0285	1				
BS	−0.0365	0.2105**	−0.0539	1			
%FD	−0.1715*	0.0513	0.0851	0.0001	1		
%ID	0.1187	−0.2112**	−0.175**	−0.4217***	−0.259**	1	
FSR	0.6158***	−0.012	0.0194	0.1279	−0.0274	0.054	1

Notes: ***, ** and * indicate significance at 0.01, 0.05 and 0.1.

The 2019 significant bivariate correlations followed a similar pattern as in 2018. As an additional correlation, the %ID showed a negative association with AGE.

The 2020 bivariate correlation followed a similar pattern to 2019. An additional correlation was that the %ID showed a negative correlation with the %FD.

### 5.5 Regression results relating to the earnings smoothness

Tables 14–16 report the regression results from the ES stepwise regression. The significance of variables with ES was similar to AQ. A difference was that AGE was positively associated with ES in 2018 and 2019. FSR showed a positive relation, and EMS showed a negative relation.

**Table 14:** Stepwise regression for earnings smoothness in 2018.

	Control variables	Plus, financial health	Plus, governance	Plus, earnings management
***Control variables***				
AGE	−0.082**	−0.079***	−0.067**	−0.069**
FSR	0.642***	0.642***	0.656***	0.634***
***Independent variables***				
EMS		−0.769***	−0.755**	−0.773**
BS			−0.372	−0.365
% ID			-4.273	-4.397
%FD			-2.060	-2.620
NDA				3.938
DA				0.754
***Statistic***				
VIF	N/A	1	1.15	1.17
Adjusted R^2^	0.210***	0.272***	0.279***	0.285***
# Observations	91	91	91	91

Notes: ***, ** and * indicate significance at 0.01, 0.05 and 0.1.

**Table 15:** Stepwise regression for earnings smoothness in 2019.

	Control variables	Plus, financial health	Plus, governance	Plus, earnings management
***Control variables***				
AGE	−0.073***	−0.065**	−0.049*	−0.051**
FSR	0.471***	0.487***	0.496***	0.492***
***Independent variables***				
EMS		−0.641***	−0.621***	−0.655***
BS			−0.360*	−0.316*
% ID			−1.241	−0.179
%FD			-3.46	-2.380
NDA				4.283
DA				−0.500
***Statistic***				
VIF	N/A	1	1.17	1.18
Adjusted R^2^	0.242***	0.329***	0.349***	0.362***
# Observations	97	97	97	97

Notes: ***, ** and * indicate significance at 0.01, 0.05 and 0.1.

**Table 16:** Stepwise regression for earnings smoothness in 2020.

	Control variables	Plus, financial health	Plus, governance	Plus, earnings management
***Control variables***				
AGE	−0.048**	−0.044*	−0.033	−0.038*
FSR	0.423***	0.427***	0.442***	0.431***
***Independent variables***				
EMS		−0.509***	−0.531***	−0.599***
BS			−0.346*	−0.348*
% ID			-4.517	-5.464
%FD			-4.856*	-4.776*
NDA				7.807
DA				−0.040
***Statistic***				
VIF	N/A	1	1.17	1.21
Adjusted R^2^	0.389***	0.475***	0.510***	0.521***
# Observations	98	98	98	98

Notes: ***, ** and * indicate significance at 0.01, 0.05 and 0.1.

### 5.6 Additional analysis

This study examined three contrasting business outlook periods. As noted in the literature review, time can influence financial reporting quality [58]. This study investigated whether ESG earnings are persistent from one period to another, comparing earnings of the current period with that of the previous period using four past years and current year observations in the regression equation [54], as shown in Eq (8). The previous period’s earnings parameter (or the slope) coefficient represents earnings persistence. This study used earnings persistence as the dependent variable and re-ran the regression models with independent and control variables, and the regression models showed no statistical significance for interpretation.


$$Earnings_{t}/TA_{t-1}=a+b*Earnings_{t-1}/TA_{t-1}+e$$ (8)

Where:

b is persistence.

The study also computed earnings predictability to determine whether past earnings can predict future earnings, calculated as the square root of the residual variance, obtaining the residual from the persistence regression model [30] as shown in Eq (9). The study estimated the predictability values as the dependent variable with independent and control variables and ran the regression models, but found that the models were not statistically significant.


$$Predictability=square\;root\;(\sigma^{2}\;e)$$ (9)

Where:

e is the residual value obtained from the persistent measurement Eq (8);

σ^2^ is the variance of the parameter (in this case, the parameter is the residual value).

### 5.7 Hypotheses summary

Table 17 shows the hypotheses that the study has confirmed, as well as their contribution to the literature. EMS showed a negative association with AQ and ES pairwise correlations and also in the regression models. The findings show that financially healthy firms had lower AQ and ES. Financially healthy firms likely make more investments in social and environmental projects. The extended payback period means that there can be more errors in estimating accruals as receivables and payables. Managers are also prone to erroneously judge the company’s cash flow and earnings forecast [18,21,64].

**Table 17:** Summary of hypotheses.

Variable	Hypothesis	Relationship	Expected	Actual
***Independent—EMS***				
	1	EMS and AQ	Yes	Positive
	2	EMS and ES	Yes	Positive
***Independent—governance***				
	3a	BS and AQ	Nil	Nil
	3b	%ID and AQ	Nil	Nil
	3c	%FD and AQ	Nil	Nil
	4a	BS and AQ	Nil	Nil
	4b	%ID and AQ	Nil	Nil
	4c	%FD and AQ	Nil	Nil
***Independent—earnings management***				
	5a	DA and AQ	Nil	Nil
	5b	NDA and AQ	Nil	Nil
	6a	DA and ES	Nil	Nil
	6b	NDA and ES	Nil	Nil
***Control—age***				
		AGE and AQ		Nil
		AGE and ES		Positive
		FSR and AQ		Positive
		FSR and ES		Positive

Where

AQ is the accrual quality

ES is the earnings smoothness

EMS is the emerging markets score

BS is the board size

%ID is the percentage of independent directors on the board

%FD is the percentage of female directors on the board

DA is the discretionary accruals

NDA is the nondiscretionary accruals

FSR is the firm-specific risk

AGE is the firm age.

However, these investments delay financial returns and create a broader gap between earnings and cash flows from operations [86,87]. The ESG firms had not engaged in ES to ease it. It is possible that ESG firms are more ethical and do not engage aggressively in ES. The low ES mean and median numbers in the descriptive statistics support this conclusion. The findings from this study regarding ESG firms listed in China contradict the literature that has reported that Chinese firms commonly practice earnings management [59].

Firm-specific risk shows the variability in firms’ accounting performance based on return on assets, and it significantly influences AQ and ES. Although no conclusive literature exists, the social and environmental project investments undertaken by ESG firms can contribute to earnings volatility [62].

In ESG firms, governance functioning has little influence on AQ and ES. Governance showed no effect on AQ and ES. It is likely that governance functions primarily to take care of shareholder interests. Another possibility is that ESG firms invest in social and environmental projects that take time to provide sufficient financial returns, or that provide lower returns than conventional investments [67].

## 6. Conclusion

This study contributes to the literature by investigating the status of hypotheses relating to ESG firms’ financial health/distress, governance and earnings management, and their causal relationship with financial reporting quality. The study examined three different business outlooks for the same firms, which can have three systematic risks. The study contributes to the literature by showing that the financial reporting quality determinants have had no effect on systematic risks and have behaved similarly across those periods. Additionally, the study found that ESG firms can contain firm-specific risks and have more volatile earnings, making earnings less persistent and predictable. ESG firms’ financial health positively influenced financial reporting quality, but governance and earnings management had no influence. This is the first study to investigate ESG firms listed in China and find the chosen determinants’ influence on financial reporting quality. These findings have theoretical and managerial implications, which are outlined below, along with future research directions.

### 6.1 Theoretical implications

Altman’s Z-score model is a valuable tool to ascertain the financial health of ESG firms. It supports the fact that ESG firms follow stakeholder capitalism. As the financial health of ESG firms improves, they are more likely to increase their investment in projects that meet the ESG criteria. The findings also showed that ESG firms have not opportunistically managed earnings. Agency theory points out that managers have not prioritised their self-interest to gain personal financial benefits from ESG firms. The sample ESG firms listed in China are more aligned with stakeholder capitalism and less aligned with agency theoretical perspectives.

The results support the cultural stakeholder theory whereby social cultural conditions are such that stakeholders, including shareholders, trust ESG firms to behave ethically. Deriving the meaning from Hofstede’s cultural dimensions, societal cultural perspectives of firm value creation are different [65]. Chinese societal culture is highly interdependent and long-term oriented. Although governance did not have a significant influence on financial reporting quality, the managerial behaviour of ESG firms shows that they have not engaged in earnings management, and have made greater investments in projects meeting ESG criteria with increasing financial health. ESG firms that build long-term trustworthy relationships with stakeholders support the societal cultural dimension of long-term orientation in Chinese society [19,48].

### 6.2 Managerial implications

This study contributes to the understanding that ESG firms that are listed in China and invest in social and environmental projects give rise to structural delays in converting earnings into operating cash flows. ESG firms have this time delay as a structural feature as a result of sustainability-driven investments. It is difficult to narrow the gap between reported earnings and cash flows from operations through accruals adjustments and using managerial judgement to smooth earnings with information that is unknown to shareholders. ESG firms can only do so by becoming unethical or breaking the law. The results show that ESG firms are ethical because they do not engage in earnings management. Earnings smoothness also has low values, which shows that ESG firms do not aggressively use managerial discretion to adjust earnings [88].

Research that is unrelated to ESG has shown that effective corporate governance contributes to financial reporting quality [31,52,89]. The board can do little to rectify ESG firms’ structural gap between earnings and cash flows from operations. The monitoring and compliance mechanisms that are applicable in a typical principal–agency relationship—primarily fiduciary functions—cannot overcome the structural gap between earnings and cash flows from operations. In these situations, an effective way to increase financial reporting quality is for managers to make judgments with negligible bias and errors.

This study found that the increasing financial health of ESG firms is likely to encourage them to invest more in social and environmental projects. This will magnify the delay between earnings becoming operating cash flows, thereby decreasing the financial reporting quality, determined by accrual quality and earnings smoothness. Managers must be conscious of such implications because they can affect the firm’s dividend payouts and shareholder interest [20].

### 6.3 Future research

The research findings are generalisable to the target population. Future research could expand the sample size for a broader firm population representation, because research shows that leaders’ capabilities influence ESG practices [21]. Although capital markets facilitate better risk sharing and market efficiency, various contextual barriers in countries can limit their benefits [7]. Replicating the study in other countries’ capital markets can broaden the understanding of ESG firms’ financial reporting quality. The empirical investigation selected the variables of financial health, governance and earnings management. Future research could broaden the variables included within the limits of model parsimony.

Animal or human ethics approval: The study did not require ethics approval because it used secondary data and had no animal or human intervention in the research project.
